# Veterinary Department, University of Pennsylvania

**Published:** 1895-07

**Authors:** 


### Veterinary Department, University of Pennsylvania.

Out of a class of twenty-one a class of eighteen was graduated in June. Many of the first- and second-year boys failed in the very severe examinations exacted of them. Out of the fifty free scholarships granted to graduates of the city High School of Philadelphia by the University of Pennsylvania, three for each of the coming two years will be available in the Veterinary Department. The commencement exercises were made more pleasant by a reunion of the graduates around the banquetting table at the Bingham House, Philadelphia.

---

The written examinations of the Royal College of Veterinary Surgeons in Scotland were held in Edinburgh and Glasgow simultaneously with those held in London, on Monday, May 13th; 190 candidates were present in Edinburgh, and 65 were present in Glasgow.

In Class C of final examination 79 candidates presented themselves in Edinburgh, and of these 36 passed; while in Glasgow 18 presented themselves and 10 passed. With the exception of one gentleman, who was not of age, all those who passed were admitted members of the Royal College of Veterinary Surgeons.

The Faculty of the Kansas City Veterinary College contains twelve veterinarians and six medical doctors, while two of the veterinarians are also M.D. graduates.

The Indiana Veterinary College has undergone a reorganization in its Board of Trustees and Faculty. E. L. Booth, M.D., and John W. Claypool have been succeeded by Drs. George H. Roberts and H. R. Macaulay as Trustees.

The following additions have been made to the Faculty: J. F. Roe, D.V.S., Anatomy, succeeding W. B. Craig, V.S.; C. Jones, M.D., Physiology, succeeding Frank Wynn, M.D.; C. J. Bell, V.S., Surgery, succeeding M. Y. Shaeffer, D.V.S.; W. Klotz, V.S., Obstetrics and Animal Castration, succeeding F. A. Balser, V.S., and L. A. Greiner, V.S. Lameness and Shoeing have been added. George E. Hunt, D.D.S., M.D., General Pathology and Morbid Anatomy; and J. O. Greeson, V.M.D., Hygienic Breeding and General Management of Domestic Animals, have been dropped from the roll of lecturers. R. Ebbitt, M.R.C.V.S., connected with the Meat Inspection Department of the Bureau of Animal Industry, has been added to the corps of lecturers on Meat and Meat Inspection, this branch having been formerly associated with the Chair of Cattle Pathology and Contagious Diseases, and filled by T. L. Armstrong, D.V.S.

Two features of the school still remain that we wish were otherwise—that of the early age (seventeen years) that students are admitted, and the two years' curriculum of scarcely six months each, the time for examinations deducted. The entrance examination remains the same.

---

We are glad to learn of the return to his first love, that of the veterinary profession, of Dr. C. H. Magill, of Philadelphia, who for the past two years has been engaged in commercial pursuits.

Dr. R. S. Huidekoper, of 154 East Fifty-seventh Street, New York City, has opened an office and infirmary at 20 Weaver Street, Newport, R. I., for the summer season.

Among those who received the degree of M.D. at the recent commencement of the Medical Department of the University of Pennsylvania was John J. Rectenwald, V.M.D., Pittsburg, Pa., a graduate in veterinary science from the Veterinary Department.

